# A novel role for APOBEC3: Susceptibility to sexual transmission of murine acquired immunodeficiency virus (mAIDS) is aggravated in APOBEC3 deficient mice

**DOI:** 10.1186/1742-4690-9-50

**Published:** 2012-06-12

**Authors:** Philip H Jones, Harshini V Mehta, Chioma M Okeoma

**Affiliations:** 1 Department of Microbiology, Carver College of Medicine, University of Iowa, 51 Newton Road, Iowa City, IA 52242-1109, USA

**Keywords:** APOBEC3, murine AIDS, LP-BM5 virus, MLV, ♀: female, ♂: male

## Abstract

**Background:**

APOBEC3 proteins are host factors that restrict infection by retroviruses like HIV, MMTV, and MLV and are variably expressed in hematopoietic and non-hematopoietic cells, such as macrophages, lymphocytes, dendritic, and epithelia cells. Previously, we showed that APOBEC3 expressed in mammary epithelia cells function to limit milk-borne transmission of the beta-retrovirus, mouse mammary tumor virus. In this present study, we used APOBEC3 knockout mice and their wild type counterpart to query the role of APOBEC3 in sexual transmission of LP-BM5 MLV – the etiological agent of murine AIDs (mAIDs).

**Results:**

We show that mouse APOBEC3 is expressed in murine genital tract tissues and gametes and that genital tract tissue of APOBEC3-deficient mice are more susceptible to infection by LP-BM5 virus. APOBEC3 expressed in genital tract tissues most likely plays a role in decreasing virus transmission via the sexual route, since mice deficient in APOBEC3 gene have higher genitalia and seminal plasma virus load and sexually transmit the virus more efficiently to their partners compared to APOBEC3+ mice. Moreover, we show that female mice sexually infected with LP-BM5 virus transmit the virus to their off-spring in APOBEC3-dependent manner.

**Conclusion:**

Our data indicate that genital tissue intrinsic APOBEC3 restricts genital tract infection and limits sexual transmission of LP-BM5 virus.

## Background

Viruses that use the genital mucosa as a portal of entry into the host include HPV, HSV, hepatitis B virus, and retroviruses like SIV, MLV, and HIV. Worldwide, HIV infection is pandemic in many countries and is primarily transmitted from infected persons to their sexual partners through contact with infected semen and vaginal secretions [1-4]. The control of sexual transmission is, therefore, pivotal to curbing the HIV pandemic, especially since the genital tract may also serve as a sanctuary in which HIV undergoes selective pressures [5-8] that may result to the emergence of drug-resistant HIV variants that may be sexually transmitted [9-11]. Indeed, it is known that patients with highly active anti-retroviral therapy (HAART) - suppressed plasma viral load continue to shed viral RNA through the genital tract [12,13]. It is therefore critical to determine whether host factors can control virus replication and evolution within the male genital tract. Since we could not use human subjects for these experiments, a mouse model offers a practical alternative. Hence, we utilize the murine acquired immunodeficiency syndrome (mAIDs) model.

mAIDs is a mouse retroviral disease caused by the viral complex LP-BM5 [14]. LP-BM5 is a mixture of murine leukemia viruses comprised of the replication competent helper virus BM5e, the mink cell cytopathic focus-inducing virus, and the replication-defective virus BM5def, responsible for the immunodeficiency [15,16]. Infection of susceptible mice with LP-BM5 causes severe immunodeficiency syndrome similar to human AIDS [14,17]. Some of the similar characteristics between mAIDS and hAIDS include hypergammaglobulinemia, lymphadenopathy, severely depressed T- and B-cell responses to mitogens, increased susceptibility to infection, disease progression and the development of B-cell lymphomas and splenomegaly [18-22]. Despite the similarities between mAIDS and hAIDS, differences between the two infectious diseases exist. Unlike human AIDS, mice suffering from mAIDS develop mediastinal lymph node enlargement and may die of respiratory failure [23]. Propagation of mAIDS in mice requires the matrix (MA) and p12 of the Gag gene of BM5def [24]. Due to the similarity in virus transmission and disease outcome between LP-BM5- and HIV infections, mAIDs is a good model for examining sexual transmission of HIV and have been used for the initial evaluation of new drugs [25-29].

To be transmitted sexually, HIV and LP-BM5 will have to overcome host resistance in genital mucosal surfaces which are composed of different types and layers of epithelial cells and mucosa-associated lymphoid tissues (MALTs). The male and female genital mucosal layers are filled with cells of the immune system, including dendritic cells (DCs), macrophages and lymphocytes [30-36]. The immune cells in the genital mucosal surfaces express pattern recognition receptors (PRRs) such as Toll-like receptors, RIG-I-like receptors, and NOD-like receptors [37-40] that function to recognize different pathogen associated molecular patterns (PAMPs) and stress signals leading to activation of the host innate and adaptive immune responses. Aside from innate and adaptive immune responses elicited by cells in the genital mucosal surfaces, the expression of anti-viral genes such as APOBEC3 (A3) in male reproductive organs has been reported [41,42].

A3 proteins are virus restriction factors that encode DNA-editing enzymes. The human genome encodes 7 A3 genes (A3 –A, -B, -C, -D/E, -F, -G, -H), and the mouse genome encodes only 1 A3 gene [43]. A3 proteins are variably expressed in hematopoietic and non-hematopoietic cells, and in different tissues [42,44-47]. The most studied human A3 genes (A3G and A3F) and mouse A3 (mA3) are reported to confer intrinsic immunity to HIV-1 and other viruses including MMTV and MLV [45-52]. In the absence of HIV-1 viral infectivity factor (Vif), A3F and A3G are packaged into progeny virions via interactions with the nucleocapsid (NC) protein and viral RNA. A3 proteins then inhibit infection in target cells by deaminating deoxycytidine residues on the DNA minus strand following reverse transcription and inducing mutations in newly synthesized HIV-1 DNA. In addition, A3F and A3G degrade reverse transcribed viral DNA prior to integration and induce mutation of viral genes in the integrated provirus [53-56]. However, in the presence of Vif, A3F and 3G are marked for proteasomal degradation [57-59], thus preventing their packaging into progeny virions; resulting in productive infection of target cells. Furthermore, there is growing evidence that A3 proteins can restrict HIV and other viral infection in a deamination independent manner [45,46,60,61]. We previously showed that endogenous mA3 restricts MMTV infection *in vivo* in a deamination independent mechanism [45]. In addition to limiting virus replication, we recently demonstrated that the rate of milk-borne transmission of MMTV was significantly increased in mice harboring targeted mutation in the mA3 gene [47], demonstrating that A3 proteins play a role in containing milk-borne virus transmission.

In the present study, we define the role of A3 in sexual transmission of retroviruses using the mAIDs model and mice with targeted mutation in the mA3 gene. Our data indicate that mice with targeted mutation of APOBEC3 gene have a higher rate of sexual transmission compared to the wild type control, suggesting a role for APOBEC3 in limiting sexual transmission.

## Results

### mA3 is expressed in genital tract tissues and gametes

**Figure 1:**
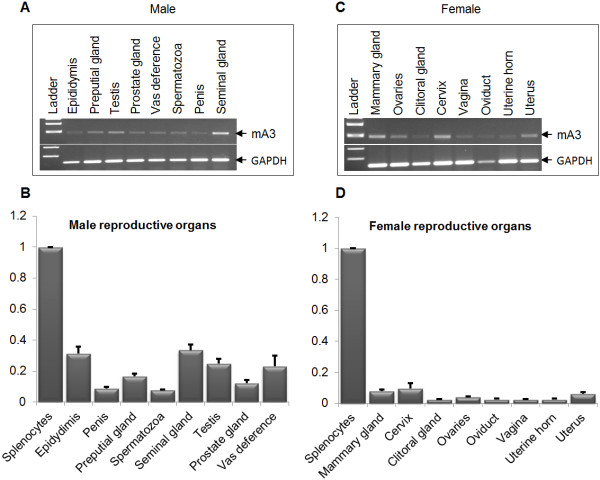
**mA3 is expressed in murine female and male reproductive organs.** Age-matched male and female WT mice on C57BL/6 background were sacrificed. At necropsy, tissue samples were obtained for RNA extraction. RNA was reverse transcribed and the resulting cDNA was used to examine levels of mA3 transcript by endpoint PCR (**A** and **C**) and real-time quantitative PCR (**B** and **D**). Data were normalized to GAPDH and presented as relative levels to splenic mA3 mRNA. Error bars are standard error; * is significance with p value equal or less than 0.05; and ** is significance with p value equal or less than 0.01. Experiment was performed 3 times with similar results.

Healthy female and male C57BL/6 mice were euthanized, and different genital tract tissues and gametes were obtained for analysis of mA3 mRNA levels by real-time quantitative PCR (qPCR). We observed that mA3 is expressed at varying levels in male (Figure 1A and 1B) and female (Figure 1C and 1D) genital tissues and gametes. In males, the epididymis and seminal glands have the highest level of mA3 expression, while the prostate gland, penis and spermatozoa have lower levels of mA3 expression (Figure 1B). The distribution of mA3 in female genital tissues shows that the cervix and mammary gland have the highest levels of expression while the oviduct, clitoral gland, uterine horn, and vagina express lower levels of mA3 mRNA (Figure 1D). Although both male and female reproductive organs express mA3 mRNA, male organs such as epididymis, seminal gland, testis, and vas deference make more mA3 transcripts than any female organ tested.

### mA3-deficient mice have increased susceptibility to acute LP-BM5 infection

**Figure 2:**
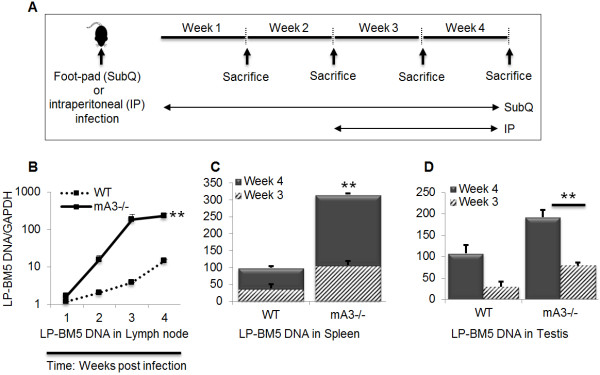
**Mice deficient in APOBEC3 gene are more susceptible to acute LP-BM5 virus infection.** Age and weight-matched WT and mA3-/- mice on C57BL/6 background (n = 5) were inoculated with LP-BM5 virus subcutaneously (SubQ) on the right hind footpad or via intraperitoneal (IP) route (**A**). Mice were sacrificed at different time frames as shown on the figures. At necropsy, lymph nodes were collected from SubQ infected mice, while spleens and testes were obtained from IP infected mice. DNA was isolated from the different tissues and subjected to quantitative PCR (qPCR) to determine level of LP-BM5 infection in lymph nodes (**B**) spleen (**C**), or Testes (**D**). All qPCR data were normalized to GAPDH and presented as relative levels. Error bars are standard error; * is significance with p value equal or less than 0.05; and ** is significance with p value equal or less than 0.01. Experiments were repeated five different times with similar results.

Since male genital organs and gametes express mA3, and since we previously showed that mA3 deficient mice are more susceptible to MLV and MMTV [45,46,51,52], we hypothesize that mice lacking the mA3 gene (mA3-/-) will have higher virus load upon infection with LP-BM5 MLV. To test this hypothesis, we inoculated wild-type (WT) and mA3-/- mice with LP-BM5 virus subcutaneously (SubQ) on the footpad or intraperitoneally (IP). Footpad infected mice were sacrificed weekly for 4 weeks while IP infected mice were sacrificed at weeks 3 and 4 (Figure 2A). Spleen and testes (IP infected) or lymph nodes (SubQ infected) were used to examine the level of infection by qPCR. We detected significantly higher levels of LP-BM5 DNA in the lymph nodes of mA3-/- mice compared to the WT controls (Figure 2B). Lymph nodes were infected as early as 1 week post inoculation, and virus load increased in a time dependent manner. Examination of the spleen (Figure 2C) and testes (Figure 2D) for proviral DNA at 3 and 4 weeks after infection showed a significant and time dependent increase in virus load in mA3-/- mice compared to the WT. We did not observe spleen or testes infection at weeks 1 and 2 following inoculation. To determine whether mA3 edits LP-BM5 genome, we generated and analyzed sequences of LP-BM5 from total cellular DNA obtained from spleens and testes of infected male mice. Analysis of LP-BM5 sequence data showed no evidence of hypermutation (data not shown).

### mA3 deficiency exacerbates mAIDs-associated lympho-proliferation and splenomegaly

**Figure 3:**
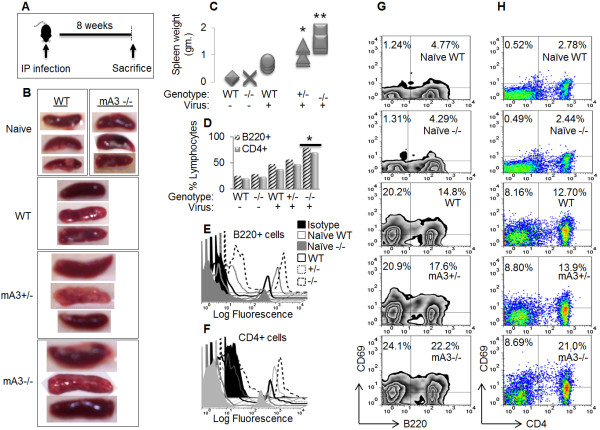
**Lympho-proliferation and splenomegaly are more pronounced in mA3 deficient mice.** Age and weight-matched WT, mA3+/-, and mA3-/- male mice on C57BL/6 background (n = 5) were inoculated with PBS (negative control) or LP-BM5 virus via the IP route (**A**). Eight weeks after inoculation, mice were sacrificed. Spleen sizes (**B**) and weights (**C**) were recorded for each genotype. Single cell suspension was obtained from splenocytes and stained with cell surface antibodies to differentiate B cells from CD4 + T lymphocytes and determine lymphocyte number using flow cytometry. B cell percentage was detected with anti-B220 (**D** and **E**), and CD4 + T percentage was detected with anti-CD4 (**D** and **F**). Activation of CD69 expression on B cells (**G**) and CD4+ T cells (**H**) was detected upon triple-staining with anti -B220, -CD4, and -CD69. Flow cytometry data were analyzed with Flowjo. Error bars are standard error; * is significance with p value equal or less than 0.05; and ** is significance with p value equal or less than 0.01. Experiments were repeated at least three different times with similar results and representative data presented.

Since C57BL/6 mice infected with LP-BM5 develop chronic splenomegaly, we predicted that loss of mA3 will result in severe splenomegaly. Age and weight matched WT, mA3+/-, and mA3-/- mice infected with LP-BM5 (Figure 3A) were examined for the development of splenomegaly. In general, gross appearance and size of the spleen were variable. mA3-/- mice show severe splenomegaly because their spleen sizes and weights were profoundly larger compared to the heterozygotes and WT controls in that order (Figure 3B and 3C). Although the spleens of infected mA3-/- mice were much larger than that of the WT, the WT spleen weights were significantly higher compared to the naïve mice (Figure 3B and 3C). Since most of the cells infected by LP-BM5 virus belong to the lymphocyte lineage [62,63] and the infected B and T cells greatly expand [62-65], we examined the percentage of B220+ and CD4+ expressing splenocytes using flow cytometry. LP-BM5 infection caused significant increase in B220 and CD4 expressing cells in mA3-dependent manner (Figure 3D to 3F). The percentage of B220 splenocytes were 25.8 ± 0.1; 29.1 ± 0.4; 48.1 ± 5.01; 57.0 ± 3.22; and 79.5 ± 6.34 in naïve WT, naïve mA3-/-, WT, mA3+/-, and mA3-/- mice in that order (Figure 3D). A similar observation was made upon examination of CD4+ T cells. The percentage of CD 4+ T cells increased from 38.6 ± 1.8 in WT mice to 50.7 ±4.4 in mA3-/- (Figure 3D). We also evaluated the expression of CD69 by B220+ B and CD4+ T cells after infection with LP-BM5. The percentage of B220+ cells expressing CD69 expanded from 4.77%/4.29% in naïve WT/mA3-/- mice to 14.8%, 17.6%, and 22.2% in WT, mA3+/-, and mA3-/- infected mice in that order (Figure 3G). A similar observation was made on CD4+ T cells expressing CD69. The percentage of CD4+ T cells expressing CD69 increased from 2.78%/2.44% in naïve WT/mA3-/- mice to 12.70%, 13.9%, and 21.0% in infected WT, mA3+/-, and mA3-/- mice respectively (Figure 3H). The expansion of CD69 expressing B220+ and CD4+ T cells has been previously reported in mice chronically infected with the LP-BM5 related retroviruses, Friend MLV [66] and MMTV [67].

### Mice lacking mA3 gene have higher genital infection and seminal plasma virus load

**Figure 4:**
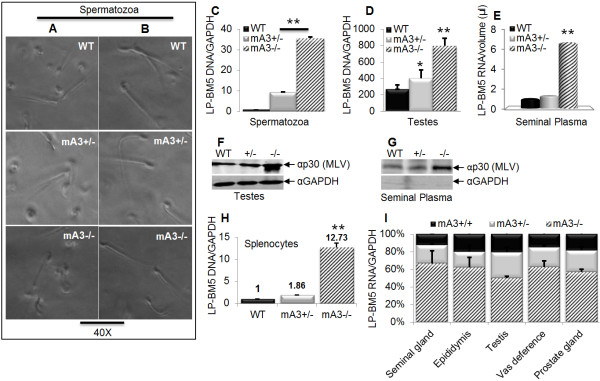
**mA3-/- mice have higher genital infection and more seminal plasma virus load.** Age and weight-matched WT, mA3+/-, and mA3-/- male mice on C57BL/6 background (n = 5) were inoculated with LP-BM5 virus via the IP route. Eight weeks after infection, mice were sacrificed, and all male reproductive organs obtained. Samples were pooled based on genotype. Spermatozoa and seminal plasma were extracted from pooled epididymis, and spermatozoa examined for morphology prior to purification (**A**). Following purification, sperm cells were also examined for morphology (**B**). DNA was isolated from spermatozoa (**C**) and testes (**D**) and evaluated for level of infection by qPCR. Total RNA was isolated from a portion (200 μl) of cell-free seminal plasma virus preparation. Equivalent concentration of RNA was reverse transcribed, and the resulting cDNA subjected to qPCR examination of viral RNA (**E**). A portion of testes (**F**) and cell-free seminal plasma virus preparation (**G**) were subjected to protein extraction and Western blot with antibodies against MLV capsid (α-p30) and murine GAPDH (α-GAPDH -used to demonstrate that the cell-free seminal plasma virus preparation devoid of cellular extract). The remaining portion of cell-free seminal plasma virus preparation was used to infect splenocytes from WT C57BL/6 *ex vivo* for 24 hours. Infected splenocytes were used for DNA extraction and qPCR examination of level of infection (**H**). Total DNA was also isolated from different male reproductive organs. Following reverse transcription of RNA, the level of viral RNA in these tissues was determined by qPCR (**I**). Western blot and qPCR data were normalized to GAPDH. Error bars are standard error; * is significance with p value equal or less than 0.05; and ** is significance with p value equal or less than 0.01. Experiments were performed at least three different times and similar results were obtained.

The presence of HIV-1 virions in human seminal plasma and seminal vesicles has been demonstrated [68-72]. To examine the impact of mA3 on virus titre and the link to sexual transmission, we examined viral load in the testes, purified spermatozoa and seminal plasma obtained from the epididymis of WT, mA3+/-, and mA3-/- mice. Upon evaluation of sperm cells, no differences in cell morphology were observed prior to sperm purification (Figure 4A). We also did not observe any difference in the morphology of spermatozoa after purification (Figure 4B). However, sperm cells from mA3-/- mice were significantly infected with LP-BM5 virus compared to the heterozygote and WT controls (Figure 4C). A similar observation was made in testicular DNA obtained from mA3-/- mice (Figure 4D). A comparison of viral RNA (vRNA) in cell-free seminal plasma as measured by qPCR showed high level of vRNA present in seminal plasma of mA3-/- (Figure 4E). To confirm that LP-BM5 MLV is present in genital tissues and seminal plasma, we used Western blot to detect viral capsid protein (p30) in these samples. The testicular cells and seminal plasma of mA3-/- mice have higher viral p30 protein (Figures 4F and 4G) compared to mA3+/- and WT counterparts. There appears to be a different banding pattern in mA3-/- lane for αp30. This banding pattern was seen with each experiment, but it is not clear why the difference and if the band is a doublet or a larger amount of the p30. Regardless, the high viral RNA and protein observed in mA3-/- mice indicate that these mice are more highly infected, and that their seminal plasma contains more viruses. The empty GAPDH blot in figure 4G indicates that the virus prepared from the seminal plasma is devoid of cellular contamination. To determine whether high seminal plasma vRNA in mA3-/- mice reflects the infectiousness of virus in the semen, we infected WT splenocytes with a portion of purified cell-free seminal plasma virus for 24 hours. Seminal plasma virus from mA3-/- was significantly more infectious than the mA3+/- and WT controls because splenocytes that were infected with the mA3-/- virus have about 11 and 12 times more LP-BM5 DNA than the mA3+/- and WT controls respectively (Figure 4H). Examination of other genital organs also reveal that mA3-/- mice harbor significantly higher viral load (vRNA) compared to the mA3+/- and WT controls (Figure 4I).

### Loss of APOBEC3 gene facilitates efficient sexual transmission of LP-BM5

**Figure 5:**
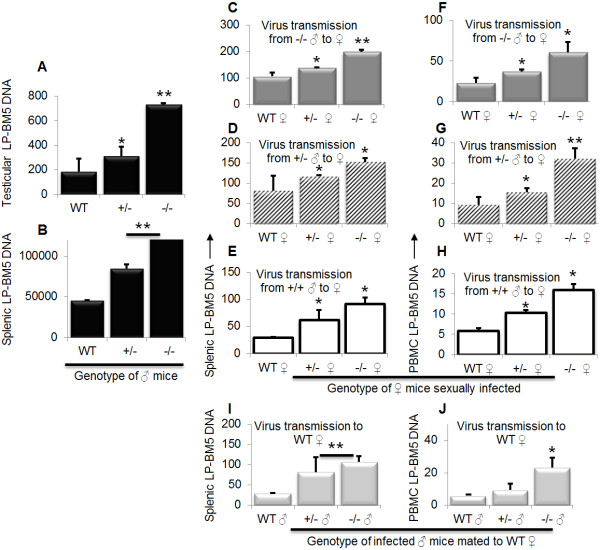
**Loss of APOBEC3 gene enhances sexual transmission of LP-BM5 virus.** Age and weight-matched WT, mA3+/-, and mA3-/- male (♂) mice on C57BL/6 background (n = 5) were inoculated with LP-BM5 virus via the IP route. Four weeks after infection, mice were mated for 6 days (normal estrus cycle in laboratory mouse) with age and weight-matched WT, mA3+/-, and mA3-/- female (♀) mice on C57BL/6 background (n = 3). Female mice were removed from their male partners, housed separately, and pregnancy status determined to ensure mating. Male mice were sacrificed upon removal of female partners (five weeks post infection). All female mice were bled on day 12 following mating to obtain peripheral blood mononuclear cells (PBMCs) and then sacrificed. Upon necropsy, the testes and spleen were collected from male mice and spleen from female mice. DNA was extracted from all samples. Rate of male mice infection was examined by examining level of viral DNA present in testes (**A**) and spleen (**B**). Rate of virus transmission was determined by evaluating level of viral DNA present in spleen (**C** to **E**) and PBMCs (**F** to **H**) of female mice of different genotypes mated to male mice of different genotypes. Side-by-side comparison of sexual virus transmission from WT, mA3+/-, or mA3-/- infected male to a WT female is shown for spleen (**I**) and PBMCs (**J**). Error bars are standard error; * is significance with p value equal or less than 0.05; and ** is significance with p value equal or less than 0.01. Experiments were performed at least three different times with similar results. ♂ = male, ♀ = female.

Previous studies have shown that murine genital organs are susceptible to LP-BM5 infection and that the virus is transmitted sexually [73]. To evaluate whether mA3 plays a role in sexual transmission of LP-BM5 from male to female mice, we infected age-matched male WT, mA3+/-, and mA3-/- mice and mated infected mice with naïve females of different genotypes. Examination of testicular and splenic DNA reveals that all male mice were infected. However, higher levels of infection in the testes (Figure 5A) and spleen (Figure 5B) were observed in mA3-/- mice compared to their mA3+/- and WT counterparts. Furthermore, infected male mice transmitted the virus to their female partners of different genotypes as evidenced by the presence of LP-BM5 DNA in spleen (Figures 5C to 5E) and PBMCs (Figures 5F to 5H) of naïve females mated with infected mice. Rate of virus transmission was significantly higher in female mice mated with mA3-/- males irrespective of the female mice genotype (Figure 5C and 5F), followed by females mated with mA3+/- mice (Figure 5D and 5G), then females mated with WT (Figure 5E and 5H). Since rate of LP-BM5 replication is highest in mA3-/- mice (Figures 4E and 4F), we examined the impact of higher virus replication on sexual transmission. Examination of virus transmission from WT, mA3+/-, or mA3-/- infected male to WT females reveals that indeed, mA3-/- mice harbor significantly more virions compared to the mA3+/-, and WT counterparts in that order (Figure 5I and 5J). This result confirms that high rate of LP-BM5 replication in mA3-/- mice translates to high rate of sexual virus transmission.

### Female mice sexually infected with LP-BM5 transmit the virus to their off-spring in APOBEC3-dependent manner

**Figure 6:**
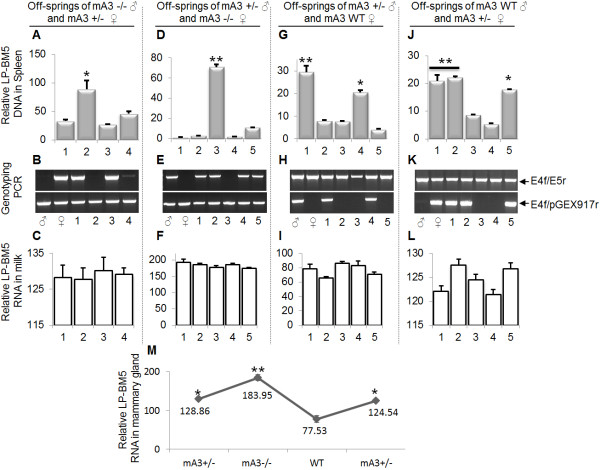
**Susceptibility of off-spring of sexually infected mothers to LP-BM5 is APOBEC3-dependent.** Age and weight-matched WT, mA3+/-, and mA3-/- male (♂) mice on C57BL/6 background (n = 5) were inoculated with LP-BM5 virus via the IP route. Four weeks after infection, mice were mated (Table 1) for 6 days with age and weight-matched WT, mA3+/-, and mA3-/- female (♀) mice on C57BL/6 background (n = 3). Female mice were removed from their male partners, housed separately throughout the gestation period. Upon parturition, mice were allowed to nurse the off-spring for 3 days and female mice and her off-springs were euthanized. The spleens of all mice (male, female, and off-spring) were collected. Mammary gland tissues from all female mice were also collected, and milk from individual off-spring was obtained as previously described [47]. DNA was extracted from spleen and used to determine level of off-spring infection (**A, D, G, J**) and genotype of all mice (**B, E, H, K**). Total RNA was extracted from milk samples and mammary gland tissues and used to determine milk viral load (**C, F, I, L**) and mammary gland viral load (**M**). Error bars are standard error; * is significance with p value equal or less than 0.05; and ** is significance with p value equal or less than 0.01. Experiments were performed three different times with similar results. ♂ = male, ♀ = female.

mA3 mediates milk-borne transmission of MMTV from an infected female to her off-spring [47]. To validate the role of mA3 in sexual transmission of LP-BM5 virus, we examined rate of virus infection in the off-springs of female mice that were sexually infected by their male partners (Table 1). We found that off-springs of sexually-infected female mice of different genotypes are infected and that level of infection depends on the presence or absence of mA3 gene. Thus, mA3-/- off-springs (Figure 6A bar 2 and 6B lane 2) from mA3-/- male and mA3+/- female (Figure 6B ♂ and ♀) show significantly higher levels of infection compared to their mA3 +/- litter mates (Figure 6A bars 1, 3, 4 and 6B lanes 1, 3, 4). Similar observations were made on 1) mA3-/- off-spring (Figure 6D bar 3) from mA3+/- male and mA3-/- female (Figure 6E ♂ and ♀) compared to its mA3 +/- litter mates (Figure 6D bars 1, 2, 4, 5 and 6E lanes 1, 2, 4, 5); 2) mA3+/- off-spring (Figure 6G bars 1, 4 and Figure 6H lanes 1, 4) from mA3+/- male and mA3+/+ female (Figure 6H ♂ and ♀) compared to their mA3 +/+ litter mates (Figure 6G bars 2, 3, 5 and Figure 6H lanes 2, 3, 5); and 3) mA3+/- off-spring (Figure 6J bars 1, 2, 5 and Figure 6K lanes 1, 2, 5) from WT male and mA3+/- female (Figure 6K ♂ and ♀) compared to their WT litter mates (Figure 6J bars 3, 4 and Figure 6K lanes 3, 4). To confirm that these differences were off-spring-mA3 gene dependent, we examined the level of vRNA in milk consumed by each off-spring from the same sexually infected female. Our results indicate no differences in milk vRNA among off-springs from the same female (Figures 6C6F6I6L); however, differences abound in the amount of vRNA that females of different genotype harbor in their mammary gland tissues, and these differences correlate with the female mA3 genotype (Figures 6M). The fact that off-springs of sexually infected female mice were infected serve as a validation of sexual virus transmission from an infected male to the female counterpart. Variation in infection level observed between pups from different parents could stem from a number of factors, including genetic disposition of the pup with respect to different genes or related factors, amount of virus in mother’s milk, rate of virus acquisition by individual pups - which could be influenced by the amount of milk consumed by the pup, as well as the amount of virus in mothers milk.

**Table 1:** mA3 genotype permutation for the generation of off-springs from parents of different genetic backgrounds

**Male (**♂**)**	**Female (**♀**)**	**Off-spring mA3 genotype**				
**mA3 genotype**	**mA3 genotype**	**1**	**2**	**3**	**4**	**5**
♂ 159 -/-	♀ 159C +/-	+/-	-/-	+/-	+/-	
♂ 160 +/-	♀ 160C -/-	+/-	+/-	-/-	+/-	+/-
	♀ 160D WT	+/-	WT	WT	+/-	WT
♂ 161 WT	♀ 161D +/-	+/-	+/-	WT	WT	+/-

### Genital organ mA3 determines susceptibility to sexual transmission

**Figure 7:**
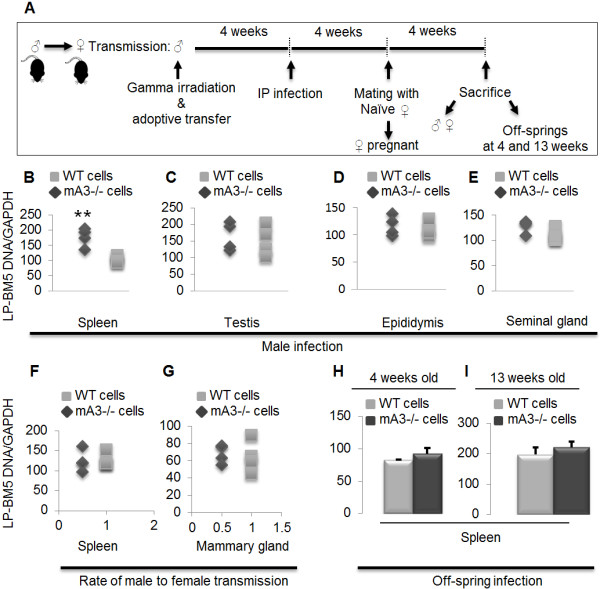
**Susceptibility of mice to sexual transmission of LP-BM5 is dependent on mA3 expressed in genitalia.** Age and weight-matched mA3-/- male (♂) mice on C57BL/6 background (n = 6) were lethally irradiated, reconstituted with bone marrow stem cells from WT or mA3 -/- mice, infected with LP-BM5 virus, and mated with naïve WT females (♀) for six days. Male mice were sacrificed at the end of mating. Female mice were allowed to nurse the resultant off-springs (heterozygotes) for three weeks. Off-springs were divided into two groups, and the first group were sacrificed at four week and the second group at thirteen weeks post-parturition (**A**). Spleen, testes, epididymis, and seminal gland were obtained from male mice and used for DNA extraction and examination of infection level by qPCR (**B** to **E**). Spleen and mammary gland tissues were obtained from female mice and used to evaluate rate of virus transmission by qPCR (**F** and **G**). Spleen tissues from the off-springs were used to examine rate of virus transmission from the infected mother (**H** and **I**). Error bars are standard error; ** is significance with p value equal or less than 0.01. This experiment was repeated 2 additional times with similar results. ♂ = male, ♀ = female.

We have shown that mA3 is expressed in male genitalia (Figure 1A and 1B) and that viral load in the genitalia organs is higher in mA3-/- mice (Figure 4F). Based on these data, we hypothesize that the higher rate of virus transmission from mA3-/- infected males to their naïve female partners is mediated by genitalia intrinsic mA3. Hence, we used adoptive transfer experiments to create mA3-/- mice that lack mA3 in the genital organs but express mA3 in their lymphocytes, and the control mice that are mA3 deficient in both genital organs and lymphocytes (Figure 7A). Results show that mA3-/- mice with WT stem cells show significantly lower splenocyte infection compared to the control mice with mA3-/- stem cells (Figure 7B), indicating that mA3-/- splenocytes are more susceptible to infection. Comparison of genital organs (testes, epididymis, and seminal gland) infection from these mice reveals no differences in level of infection (Figures 7C to 7E). To determine whether mA3 intrinsically expressed in genital organs or lymphocytes is responsible for restricting sexual virus transmission, we mated naïve WT female mice with the above males (mA3-/- males with WT or mA3-/- cells). Contrary to our observation of male infection, splenocytes and mammary gland of female mice mated with and sexually infected by males that received WT or mA3-/- cells have similar levels of infection (Figure 7F and 7G) indicating that the male genital environment controls virus transmission. Examination of the generation 1 (F1 – mA3+/-) off-springs of mA3-/- males and WT females at 4 and 13 weeks post parturition also reveal no differences in splenic viral load (Figure 7H and 7I). These genetic data suggest that genitalia-intrinsic mA3 is important and mediates restriction of sexual virus transmission.

## Discussion

HIV is mostly propagated worldwide through sexual transmission. While a number of host factors have been identified to enhance sexual transmission of HIV [74], factors that limit sexual transmission of HIV have not been described. However, APOBEC3 as an anti-HIV factor that restricts infection of hematopoietic and non-hematopoietic cells [47,50] could potentially play a role in sexual transmission. Recently, *in vivo* studies in mice show that APOBEC3 restricts MMTV infection of the lymphoid compartment, resulting in lower levels of virus infection in mammary tissue, accompanied by a decreased rate of milk-borne transmission of MMTV [47].

Here, we demonstrate a possible role for APOBEC3 in restricting a sexually transmitted virus infection. We show that mice with targeted deletion of the Apobec3 gene have higher rates of LP-BM5 infection in lymphoid and genital tissues, and that this results in enhanced rates of sexual transmission of LP-BM5 virus. It is known that LP-BM5 infects genital tissues [73] and causes severe immunodeficiency and splenomegaly [18-22]. APOBEC3 is made in genital organs; interestingly, the absence of APOBEC3 in these organs, as in APOBEC3 knockout mice, altered infection of the genital organs. Higher level of infection in APOBEC3 knockout mice provides evidence that APOBEC3 mediates the susceptibility of the genitalia and gametes to virus infection. Additionally, the significantly higher vRNA in genital organs of APOBEC knockout mice (Figure 4I), as well as high levels of extracellular vRNA and protein in seminal plasma (Figure 4E and 4G) from APOBEC3 knock mice indicates that LP-BM5 is actively replicating more efficiently in the absence of APOBEC3. Our finding is in line with published reports [75,76] which showed the occurrence of active virus replication in semen of HIV-1 infected men. The alteration of virus infection in genital compartment in APOBEC3 knockout mice positively correlates with high level of viral RNA in seminal plasma, as well as increased infectivity of seminal plasma LP-BM5 virus compared to wild type mice. The demonstration that extracellular virions from seminal plasma obtained from APOBEC3 knockout mice are more infectious than that from control mice further confirms that loss of mA3 results in higher virus load (Figure 4H). These results suggest that genital organ-intrinsic APOBEC3 expression serves to limit sexual transmission of LP-BM5, and perhaps other retroviruses. Indeed, we show that seminal fluid of APOBEC3 knock mice harbors more LP-BM5 virus compared to the wild type as visualized by Western blot (Figure 4G), but we were unable to detect APOBEC3 due to perhaps the lack of good anti-mouse APOBEC3 antibodies or limited viral protein in samples from APOBEC3 sufficient mice. The detection of LP-BM5 proviral DNA in spermatozoa and testes support previous observations made with HIV-1 and herpesvirus type 8 [77-80].

Using genetic studies, we found that APOBEC3 mediates rate of sexual transmission of LP-BM5. The different permutations of genetic backgrounds used clearly show that the APOBEC3 gene is critical in determining the outcome of sexual transmission of LP-BM5 from one partner to the other, and from a sexually infected mother to her off-spring through nursing. Although loss of APOBEC3 enhanced rate of sexual transmission, we also observed that APOBEC3 deficient female mice were more highly infected compared to the heterozygote and wild type controls in that order. These genetic experiments show that APOBEC3 also plays a crucial role in mediating virus acquisition from an infected male and that the female genetic background contributes in defining the outcome of infection.

We also used adoptive transfer and *in vivo* genetic experiments to demonstrate a clear relationship between genital-intrinsic APOBEC3 and sexual transmission. Genital-intrinsic APOBEC3 mediates control of sexual transmission since higher levels of splenocyte infection observed in male mice that received APOBEC3 knockout cells did not translate to higher rate of female partner infection. This concept was further confirmed by similar level of infection observed in the off-springs of these mice irrespective of the male parent splenocyte APOBEC3 genotype.

While murine retroviruses (beta and gamma retroviruses) are sensitive to A3 antiviral activity, they have also successfully replicated in mice. It is not clear how these infectious viruses have evaded host restriction to achieve efficient replication, since they do not encode viral accessory proteins, such as the HIV-1 Vif protein that antagonizes some human A3 proteins. However, reports of alternative mechanisms used by murine retroviruses to evade restriction by A3 proteins exist. Indeed, MLV virions have been shown to exclude mA3 [81] as well as inactivate mA3 by viral protease [82], and xenotropic murine leukemia-virus related virus (XMRV) down-regulates hA3G in prostate cancer cells [83].

Sexual transmission of HIV-1 continues to be a problem worldwide, especially in developing countries, where access to condoms is restricted by cultural and socio-economic situations. Previous studies have indicated that the use of HAART in the control of HIV transmission has mixed results since some HAART treated patients with suppressed plasma viral load continue to shed viral RNA in semen [12,13].

## Conclusion

As in immune cells and mammary epithelia cells, APOBEC3 is made in genital organs and gametes. Based on our data, we suggest that APOBEC3 expression in genital organs and gametes may have been retained as a means of inhibiting sexual transmission of viruses. In addition, APOBEC3 in genital organs could have important therapeutic implications for sexual transmission of viruses. For example, therapeutics could be developed that increase APOBEC3 level in genital organs, resulting in attenuated virions.

## Methods

### Ethics statement

Experiments involving the use of mice were performed in accordance to NIH guidelines, the Animal Welfare Act, and US federal law. Experiments were approved by the University of Iowa Animal Care and Use Committee (IACUC).

### Cells

MEF and primary testicular cells were obtained from mA3-/-, mA3+/-, and WT mice on C57BL/6 background. SC1-G6 cells chronically infected with LP-BM5 (containing ecotropic, mink cell focus-forming and defective viruses) were obtained through the AIDS Research and Reference Reagent Program, Division of AIDS, NIAID, NIH from Dr. Herbert Morse and Dr. Janet Hartley [15,19,23].

### Mice

The mA3-knockout (mA3-/-) and matched controls mA3+/-, (mA3+/+, WT) mice have been previously described [45]. All mice were housed according to the policies of the Institutional Animal Care and Use Committee of the University of Iowa.

### Adoptive transfer

Five age-matched (5 weeks old) male mice of each genotype were lethally irradiated with gamma rays. All radiation experiments were performed at the Radiation and Free Radical Research Core Facility of the University of Iowa. Mice were irradiated with a dose of 9 Gy (dose rate, 0.345 Gy/min) using a ^137^Cs source (JL Shepherd, San Fernando, CA) as previously described [47]. Four hours after irradiation, mice were reconstituted with bone marrow stem cells from either mA3-/- or WT mice. Four weeks after reconstitution, the mice were inoculated with LP-BM5 virus. Prior to infection, peripheral blood lymphocytes from each mouse were subjected to FACs analysis with conjugated anti-mouse CD8, CD4, CD11c and B220, to test for uniform reconstitution.

### Virus propagation

Cell-free LP-BM5 viral mixture was prepared from chronically infected SC-l cells as previously described [84]. Culture supernatants were collected and centrifuged at 1500 rpm for 10 minutes to clarify cellular debris. Clarified culture supernatants were filtered through a 0.45 μM filter, layered on to 20% sucrose cushion and centrifuged at 7000 rpm, 4 °C overnight. Virus pellet was resuspended in PBS at 10:1 ratio and aliquots of 100 μl stored at -80 °C until required for use.

### Cell and mouse infection

Infection of cells with cell culture or seminal plasma virus was done in triplicates in 96-well plate format. Briefly, primary testicular cells or splenocytes were plated at ~100,000 cells per well. Twenty four hours later, cells were incubated with 8 μg/ml of polybrene for 2 hours. Polybrene-containing medium was removed and replaced with 100 μl of virus-containing medium or PBS for control. Cells were spinoculated at 1200 rpm for 2 hours at room temperature. Following spinoculation, cells were incubated at 37 °C for 24 hours. DNA was extracted from cells for examination of viral load. Mice infections were done either subcutaneously (SubQ) on the hind footpad or intraperitoneally (IP). For footpad inoculation, 30 μl of virus or PBS was injected into the footpad tissue and IP inoculation was performed with 200 μl of virus or PBS. At the appropriate time depending on experiment, mice were sacrificed and relevant tissues obtained for downstream analysis.

### Genotyping mA3 -/- mice

Mice genotypes were determined using two parallel PCRs as previously described [45]. Briefly, DNA was extracted and 50 ng of genomic DNA and 25 pmol of primers were used in a total volume of 25 μl PCR reaction. PCR amplicons were separated on a 2% agarose gel.

### Nucleic acid isolation and real-time quantitative PCR

Milk and seminal plasma viral loads were determined for BM5def as previously described [85]. Briefly, total RNA was isolated from seminal fluid (200 μl) or milk (50 μl) samples using Trizol RNA Reagent (AMRESCO). Isolated RNA was DNAsed and subjected to cDNA synthesis (Applied Biosystems, ABI) as previously described [86]. DNA was also isolated from different tissues using QIAGEN-QIAamp DNA Mini Kit following manufacturer’s recommendation. Nucleic acid concentration and purity were measured spectrophotometrically at 260/280 nm. DNA and cDNA were amplified with primers and probe specific to BM5def [87] and GAPDH using ABI 7500 FAST thermal cycler (ABI).

### Flow cytometry

Approximately, 1 × 10^6^ splenocytes were stained in PBS + 1% bovine serum albumin (Sigma-Aldrich) for 30 minutes on ice with APC-, FITC-, and PE-conjugated antibodies and the resulting immunofluorescence was analyzed by use of a FACSCalibur flow cytometer (BD) to detect the expression of murine CD69, B220, and CD4 (BD or eBioscience). Cellular frequency and fluorescence intensity were determined by Flowjo analysis software (TreeStar). APC-, FITC-, and PE-conjugated Ig isotypes of irrelevant specificity were used as control for each monoclonal antibody.

### Western blot

Western blots of virus preparations and cell extracts were probed with anti-p30 (MLV capsid) and anti-GAPDH. The species-appropriate IRDye secondary antibodies were used, followed by detection with the Odyssey Infrared Imaging System (LI-COR Biosciences).

### Virus purification from seminal plasma

The seminal samples obtained from epididymis were processed immediately and pooled within genotype to provide sufficient seminal plasma volume and seminal cells. Pooled epididymal seminal samples were centrifuged for 15 minutes at 400 g. The pellet was further purified to obtain pure sperm cells as discussed below. The supernatant was collected and filtered through a 0.45 μM filter. Virions were purified from the supernatants by ultracentrifugation at 40,000 rpm for 30 minutes. Viral pellet was resuspended in PBS and stored in 50 μl aliquots at -80 °C until required for use.

### Spermatozoa purification

Pooled epididymal seminal samples were subjected to standard swim-up technique to purify sperm cells. Briefly, semen samples were centrifuged for 15 min at 400 g. The pellets were resuspended in pre-warmed 2.5 ml Ham's F-10 culture medium (Sigma) supplemented with 10% human serum albumin. Cells were layered over equivalent volume of 80% Percoll and 40% Percoll and centrifuged for 15 minutes at 400 g as previously described [88]. The supernatant was discarded and the pellet was resuspended in Ham's F-10 culture medium and thereafter centrifuged for 15 minutes at 400 g. The supernatant was removed and the pellet was overlaid with 2.5 ml Ham's F-10 medium and incubated for 60 min in 5% CO_2_ at 37 °C to allow spermatozoa to swim up from the pellet. A sterile Pasteur pipette was used to remove the supernatant containing actively motile sperms. A drop of the sample was examined under light microscope and images acquired with Nikon Eclipse Ti (Nikon Instruments). Purified cells were stored at -80 °C until required.

### Statistical analysis

The paired two-tailed Student's *t* test was used for statistical analysis and a p value of 0.05 was considered significant. Error bars represent standard deviations.

## Abbreviations

A3, APOBEC3; HIV, human immunodeficiency virus; MLV, murine leukemia viruses; mAIDS, murine acquired immunodeficiency syndrome.

## Competing interests

The authors have no conflicting financial interests.

## Authors’ contributions

CMO conceptualized and designed research, PHJ, HVM, CMO performed research, and analyzed data; PHJ, HVM, CMO wrote and read the paper. All authors reviewed the manuscript and approved the final version.
